# Expression of neuroendocrine markers predicts increased survival in triple-negative breast cancer patients

**DOI:** 10.3389/fendo.2023.1205631

**Published:** 2023-12-06

**Authors:** Chuan Xia, Songjie Shen, Junyi Pang, Longyun Chen, Jie Yan, Zhiyong Liang, Xinyu Ren

**Affiliations:** ^1^Department of Pathology, Molecular Pathology Research Center, Peking Union Medical College Hospital, Chinese Academy of Medical Science, Beijing, China; ^2^Department of Breast Surgery, Molecular Pathology Research Center, Peking Union Medical College Hospital, Chinese Academy of Medical Science, Beijing, China

**Keywords:** triple-negative breast cancer, synaptophysin, chromogranin A, neuroendocrine markers, prognosis

## Abstract

**Background:**

The significance of neuroendocrine (NE) markers in triple-negative breast cancer (TNBC) patients has not been investigated. This study aims to clarify the incidence and prognostic significance of NE marker expression in TNBC, determine its association with other clinicopathological parameters, and further explore the pathological features and potential treatment options for TNBC patients expressing NE markers.

**Methods:**

Clinicopathological data were collected from 396 TNBC patients undergoing radical breast cancer surgery at Peking Union Medical College Hospital from January 2002 to December 2014, with a final follow-up in July 2019. Immunohistochemistry (IHC) staining was performed for NE markers including chromogranin A (CgA) and synaptophysin (Syn). For TNBC patients with positive NE marker expression, IHC staining was then performed for alpha-thalassemia/mental retardation X-linked (ATRX), O(6)-methylguanine-methyltransferase (MGMT), somatostatin receptor 2 (SSTR2), and programmed death receptor-ligand 1 (PD-L1). The chi-square or Fisher exact test was used to evaluate the correlations between NE marker expression and other parameters. Survival curves were plotted using the Kaplan-Meier (K-M) method to assess the prognostic significance of NE markers in TNBC.

**Results:**

NE marker-positive staining was observed in 7.6% (30/396) of all TNBC cases. Only 0.5% (2/396) cases had ≥ 90% neoplastic cells expressing NE markers. Positive NE marker expression was associated with negative basal-like marker expression. K-M survival analysis showed that the NE marker-positive TNBC patients had higher disease-free survival (DFS) rates than the NE marker-negative patients at the same stage. Among the 30 NE marker-positive TNBC cases, 13.3% and 26.7% showed negative IHC staining for ATRX and MGMT, respectively, while 13.3% had a 3+ score for SSTR2 IHC staining. For PD-L1 IHC staining, 13.3% of the 30 TNBC cases were higher than 10 scores in Combined Positive Score (CPS), and 10.0% were higher than 10% in Tumor Cell Proportion Score (TPS).

**Conclusion:**

There was a small proportion of TNBC patients expressing NE markers. TNBC patients with positive NE marker expression had a better prognosis than the negative group at the same stage. TNBC cases with positive NE marker expression may potentially benefit from immunotherapy or somatostatin analogue treatment.

## 1 Introduction

Triple-negative breast cancer (TNBC) is a highly aggressive breast cancer subtype that has heterogeneous clinical and molecular characteristics (1, 2). Approximately 12% to 17% of breast cancer cases are TNBC, defined as tumors that lack expression of estrogen receptor (ER), progesterone receptor (PR), and human epidermal growth factor receptor type 2 (HER2) (3). The lack ER and PR expression makes it difficult for TNBC patients to benefit from endocrine therapy, while the negative expression of HER2 prevents effective targeted therapy. This results in worse prognosis for TNBC patients compared with other breast cancer subtypes. Therefore, identifying and validating novel targeted therapies and prognostic indicators for TNBC has become an urgent need for breast cancer research.

The prognostic significance of neuroendocrine (NE) differentiation in breast cancer has been controversial, partially because the classification criteria for neuroendocrine neoplasms (NENs) have evolved over the years or the limited numbers of NEN cases in breast cancer. Neuroendocrine carcinoma (NEC) of the breast was formally proposed as a distinct entity in 2003, defined as a subtype of invasive mammary carcinoma in which >50% of the tumor cells express NE markers (4, 5). The most commonly used NE markers in the breast include synaptophysin (Syn) or chromogranin A (CgA) (6, 7). The 2012 WHO Classification later stated that the 50% threshold for NE marker positivity was no longer required because of its arbitrary nature as a criterion (6). The newly published WHO Classification in 2019 emphasized that the key features of NENs are neuroendocrine histological features and expression of NE markers (8). Some cohort studies have explored the prognostic significance of NE markers in breast cancer. The majority of the studies have shown that breast cancer patients with NE features have worse prognoses compared with those without NE features (4, 9–11), while some studies have reported better prognoses (12–14) or no significant difference (9, 15–17). Currently, there were no reports about the prognosis of TNBC patients with NE marker expression.

The first-line therapy for NENs in breast cancer is mostly surgery combined with or without adjuvant radiotherapy, chemotherapy, or neoadjuvant chemotherapy. This is based on the therapeutic principles of other types of breast cancer, and the cases were treated with endocrine or targeted therapy according to the ER/PR and HER2 status. However, the relevant studies for the therapy of NENs in breast cancer are mainly case reports or case series (8, 18–22). In recent years, several studies have identified immunohistochemistry (IHC) markers that have prognostic or therapeutic significance for NENs, including ATRX, MGMT, SSTR2, and PD-L1 (23–30). However, there are no studies on therapeutic regimen for NENs in TNBC. It remains to be explored whether TNBCs with positive expression of NE markers can be treated as NENs at any other anatomical site.

Thus, we utilized two most common used NE markers, Syn and CgA, to evaluate their expression levels in TNBC patients and observe their correlations with clinicopathological parameters. Additionally, we sought to determine the prognostic significance of NE marker expression in TNBC patients and explore the potential treatment options for such individuals.

## 2 Materials and methods

### 2.1 Case enrollment and clinicopathological data acquisition

Breast cancer patients who underwent radical surgery at Peking Union Medical College Hospital from January 2002 to December 2014 and were diagnosed with TNBC (both ER and PR were negative by IHC and HER2 was 0 or 1+ score by IHC or 2+ score without gene amplification confirmed by fluorescent *in situ* hybridization (FISH)) were enrolled in the study consecutively, with final follow-up in July 2019. The exclusion criteria were as follows: (1) the amount of tumor tissue was insufficient for IHC staining; (2) the survival status and dates of recurrence or death (if any) could not be determined at the time of registration or during follow-up; (3) neoadjuvant treatment was administered before surgery; and (4) distant metastasis occurred before the patient underwent surgery. Clinicopathological data, including age, operation date, operation method, radiotherapy or chemotherapy regimens, tumor size, number of lymph nodes metastases, stage, histological type, histological grade, P53 expression, Ki-67 expression, and basal-like phenotype (defined as positive for CK5/6, CK14, and/or EGFR expression), were collected by reviewing pathology databases and medical records. Follow-up information was acquired by telephone or referring to medical records. The end points included: (1) recurrence of breast cancer (local recurrence or distant metastasis) and (2) death caused by breast cancer. Disease-free survival (DFS) and overall survival (OS) were defined as the period from the date of surgery to recurrence of breast cancer and to breast cancer-related death, respectively. The study was approved by the Ethics Committee of Peking Union Medical College Hospital and informed consent was obtained from patients.

### 2.2 IHC staining

IHC staining for NE markers, including CgA and Syn, was performed on 4 µm-thick sections of TNBC samples using a Ventana Benchmark XT autostainer (Ventana Medical Systems Inc., Tucson, AZ, USA). IHC staining for ATRX, MGMT, SSTR2, and PD-L1 (22C3) was then performed on TNBC samples that showed positive expression of NE markers. All staining steps were performed following the manufacturer’s instructions. Antibody information and the respective optimized assay conditions are listed in **Table 1**.

**Table 1:** Antibodies used for the immunohistochemistry staining.

Antibody	Clone	Dilution	Source	Positive pattern	Positive control	Heat-induced antigen retrieval	Incubation
Syn	Mouse monoclonal antibody (27G12)	Prediluted	Leica Biosystem	Cytoplasmic staining	Pancreas	100°C 30min	RT* 15min
CgA	Mouse monoclonal antibody (LK2H10)	Prediluted	ZSGB-BIO	Cytoplasmic staining	Pancreas	100°C 30min	RT 15min
ATRX	rabbit polyclonal antibody	Prediluted	Dako	Nuclear staining	Tonsil	100°C 30min	RT 40min
MGMT	SD9	Prediluted	Roche	Nuclear and/or cytoplasmic staining	Tonsil	100°C 30min	36°C 16min
SSTR2	rabbit monoclonal antibody (EP149)	Prediluted	Dako	Membrane staining	Meningioma	100°C 30min	RT 40min
PD-L1	Murine monoclonal antibody (22C3)	Prediluted	Dako	Membrane staining	Placenta	100°C 30min	37°C 16min

*RT, room temperature.

IHC slides were independently evaluated by two experienced pathologists. Both the staining intensity and percentage of positive tumor cells were recorded for Syn, CgA, ATRX, and MGMT. The SSTR2 staining results were given a score of 0, 1+, 2+, or 3+ in reference to the HER2 evaluation guidelines (31). The PD-L1 IHC staining results were interpreted by both TPS% and CPS in reference to the PD-L1 IHC 22C3 pharmDx Interpretation Manual. The average value was used when there were discrepancies between the two pathologists. Positive IHC staining for Syn and/or CgA was considered as positive for NE markers.

### 2.3 Statistical analysis

The correlation analysis between NE marker expression and other clinicopathological parameters in TNBC was performed using the chi-square or Fisher exact test. A two-sided *P*-value < 0.05 was considered statistically significant. Kaplan-Meier (K-M) survival analysis and the log-rank test were used to analyze the effects of NE markers on DFS and OS of TNBC patients. Statistical analysis was performed using SPSS 25.0 software (SPSS, Chicago, IL, USA).

## 3 Results

### 3.1 Clinicopathological characteristics of TNBC patients

A total of 455 TNBC patients were consecutively included in this cohort study, of which 32 cases were lost to follow-up. The remaining 423 cases which had partial or complete clinicopathological data were included in the statistical analysis. Among these 423 cases, 27 cases had no NE marker IHC results because of insufficient amounts of tumor tissue. These cases were excluded from further correlation analyses, leaving 396 cases to be counted.

The 423 TNBC patients were all female, with a mean age at diagnosis of 49.8 years (range: 25–90 years). The clinicopathological features are shown in **Table 2**. The histological type of 409 cases (96.7%) was invasive breast carcinoma of no specific type (IBC-NST). Additionally, 302 cases (71.4%) were grade 3 and only 1.2% were grade 1.

**Table 2:** Clinicopathological features of 423 TNBC patients.

Clinicopathological parameters	N (%)
Age (years)	
<50	222 (52.5)
≥50	197 (46.6)
NA	4 (0.9)
Surgical methods	
Modified radical mastectomy	367 (86.8)
Other	54 (12.8)
NA	2 (0.5)
Adjuvant chemotherapy	
Yes	331 (78.3)
No	55 (13.0)
NA	37 (8.7)
Adjuvant radiotherapy	
Yes	122 (28.8)
No	264 (62.4)
NA	37 (8.7)
Tumor size	
≤2cm	196 (46.3)
> 2cm, ≤ 5cm	206 (48.7)
> 5cm	20 (4.7)
NA	1 (0.2)
No. of lymph nodes metastases	
0	236 (55.8)
1-3	97 (22.9)
4-9	36 (8.5)
≥10	48 (11.3)
NA	6 (1.4)
Stage	
I	132 (31.2)
II	204 (48.2)
III	87 (20.6)
Histological type	
IBC-NST	409 (96.7)
Other	13 (3.1)
Invasive lobular carcinoma	5
Mixed IBC-NST and lobular carcinoma	3
Invasive micropapillary carcinoma	3
Micropapillary mucinous carcinoma	2
NA	1 (0.2)
Grade	
Grade 1	5 (1.2)
Grade 2	115 (27.2)
Grade 3	302 (71.4)
NA	1 (0.2)
P53	
Positive	237 (56.0)
Negative	184 (43.5)
NA	2 (0.5)
Ki-67%	
<14%	45 (10.6)
≥14%	364 (86.1)
NA	14 (3.3)
Basal-like markers	
Positive	348 (82.3)
Negative	67 (15.8)
NA	8 (1.9)
Neuroendocrine markers	
Positive	30 (7.1)
<10% of tumor cells positive for Syn and/or CgA	20
10%–89% of tumor cells positive for Syn and/or CgA	8
≥90% of tumor cells positive for Syn and/or CgA	2
Negative	366 (86.5)
Syn	
Positive	26 (6.1)
Negative	370 (87.5)
CgA	
Positive	8 (1.9)
Negative	388 (91.7)
NA	27 (6.4)
Recurrence	
Yes	138 (32.6)
No	285 (67.4)
Local recurrence	
Yes	43 (10.2)
No	380 (89.8)
Distant metastases	
Yes	108 (25.5)
No	315 (74.5)
Death	
Yes	87 (20.6)
No	336 (79.4)

IHC staining results showed that, among 396 TNBC patients with known NE marker staining results, 30 cases (7.6%) were NE marker-positive. For the percentage of immunoreactive tumor cells, 20 patients were < 10%, eight patients were 10%–90%, and two patients were ≥ 90%. In addition, 26 cases (6.6%) were Syn-positive and eight cases (2.0%) were CgA-positive. Four cases (1.0%) were both Syn-positive and CgA-positive.

The mean follow-up time was 74 months (range: 2–201 months). Recurrence occurred in 138 patients (32.6%), including distant metastasis sites such as bone, lung, brain, and liver (108 cases, 25.5%). Local recurrence sites included the chest wall, axillary lymph node, and supraclavicular lymph node (43 cases, 10.2%). Overall, 87 patients (20.6%) died of breast cancer during the follow-up period. Three patients without recurrence died of ovarian cancer, heart failure, and lung infection, respectively.

### 3.2 K-M survival analysis of the effect of NE markers on TNBC patient prognosis

The mean DFS of TNBC was 136 months (95% confidence interval (CI): 127–144), and the five-year DFS rate was 68.4% ( ± 2.3%). The mean OS was 158 months (95% CI: 151–166), and the five-year OS rate was 81.0% ( ± 2.0%).

**Figure 1:**
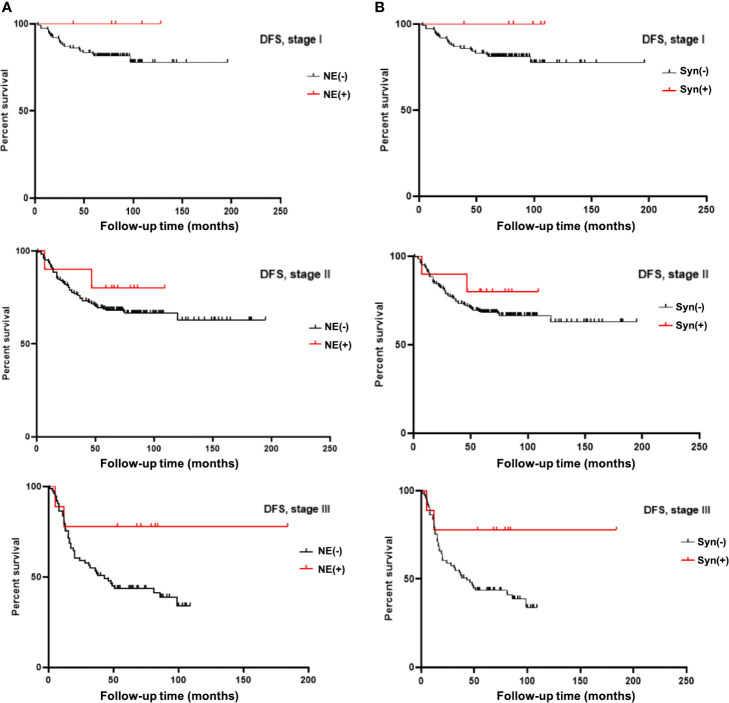
**(A)** K-M survival analysis showed neuroendocrine marker (NE) expression significantly prolongated DFS time in TNBC patients stratified by stage. **(B)** K-M survival analysis showed Syn expression significantly prolongated DFS time in TNBC patients stratified by stage.

Considering the influence of different disease stages on prognosis, we stratified the 396 TNBC patients by stage and conducted K-M survival analysis. The results are shown in **Tables 3**, **4**. The patients in the NE marker-positive group had significantly longer DFS than those in the NE marker-negative group when stratified by stage (*P*=0.040). The survival curves are shown in **Figure 1A**. No significant differences in OS were found between the two groups.

**Table 3:** K-M survival analysis of the effect of NE markers on DFS in the 396 TNBC patients stratified by stage.

	Stage	Five-year DFS rate % (standard error %)		*P* value
		Positive group	Negative group	
NE markers	I	100.0 (-)	81.5 (3.7)	0.040
	II	80.0 (12.6)	68.2 (3.4)	
	III	77.8 (13.9)	43.6 (5.8)	
Syn	I	100.0 (-)	81.3 (3.7)	0.032
	II	80.0 (12.6)	68.2 (3.4)	
	III	77.8 (13.9)	43.6 (5.8)	
CgA	I	66.7 (27.2)	82.7 (3.5)	0.718
	II	50.0 (25.0)	69.3 (3.4)	
	III	100.0 (-)	46.8 (5.6)	

**Table 4:** K-M survival analysis of the effect of NE markers on OS in 396 TNBC patients stratified by stage.

	Stage	Five-year OS rate % (standard error %)		*P* value
		Positive group	Negative group	
NE markers	I	100.0 (-)	92.7 (2.5)	0.059
	II	91.7 (8.0)	81.5 (2.9)	
	III	88.9 (10.5)	57.5 (5.9)	
Syn	I	100.0 (-)	92.8 (2.4)	0.074
	II	90.0 (9.5)	81.6 (2.9)	
	III	88.9 (10.5)	57.5 (5.9)	
CgA	I	100.0 (-)	93.1 (2.4)	0.236
	II	100.0 (-)	81.8 (2.8)	
	III	100.0 (-)	60.1 (5.6)	

Furthermore, we analyzed the effects of Syn and CgA expression on TNBC prognosis separately. The results are also shown in **Tables 3**, **4**. The DFS in the Syn-positive group were still significantly longer than those in the Syn-negative group when compared by stage (*P*=0.032). The survival curves are shown in **Figure 1B**. However, no significant differences were found between the CgA-positive and CgA-negative groups.

### 3.3 Correlations between NE markers and other TNBC parameters

The correlation analysis results for NE markers and other clinicopathological parameters in 396 TNBC patients are shown in **Table 5**. Positive NE marker expression was significantly negatively correlated with basal-like marker expression (*P*=0.004). However, we did not observe any significant correlation between NE markers and other clinicopathological features, such as patient age, tumor size, number of lymph nodes metastases, stage, grade, P53, or Ki-67.

**Table 5:** Correlation between NE markers and other parameters of the 396 TNBC patients.

Parameters	No.	NE markers		*P* value
		Positive (%) (30)	Negative (%) (366)	
Age (years)				
<50	203	16 (7.9/55.2)	187 (92.1/51.5)	0.704
≥50	189	13 (6.9/44.8)	176 (93.1/48.5)	
NA	4	1	3	
Tumor size				
≤5cm	376	27 (7.2/90.0)	349 (92.8/95.6)	0.348
> 5cm	19	3 (15.8/10.0)	16 (84.2/4.4)	
NA	1	0	1	
No. of lymph nodes metastases				
0	219	12 (5.5/41.4)	207 (94.5/57.3)	0.096
≥1	171	17 (9.9/58.6)	154 (90.1/42.7)	
NA	6	1	5	
Stage				
I, II	314	21 (6.7/70.0)	293 (93.3/80.1)	0.191
III	82	9 (11.0/30.0)	73 (89.0/19.9)	
Grade				
Grade 1, 2	109	9 (8.3/30.0)	100 (91.7/27.4)	0.759
Grade 3	286	21 (7.3/70.0)	265 (92.7/72.6)	
NA	1	0	1	
P53				
Positive	223	20 (9.0/66.7)	203 (91.0/55.8)	0.247
Negative	171	10 (5.8/33.3)	161 (94.2/44.2)	
NA	2	0	2	
Ki-67%				
<14	41	4 (9.8/13.3)	37 (90.2/10.5)	0.859
≥14	342	26 (7.6/86.7)	316 (92.4/89.5)	
NA	13	0	13	
Basal-like markers				
Positive	330	20 (6.1/66.7)	310 (93.9/86.4)	0.004*
Negative	59	10 (16.9/33.3)	49 (83.1/13.6)	
NA	7	0	7	

### 3.4 Pathological characteristics of the 30 NE marker-positive TNBC cases

**Figure 2:**
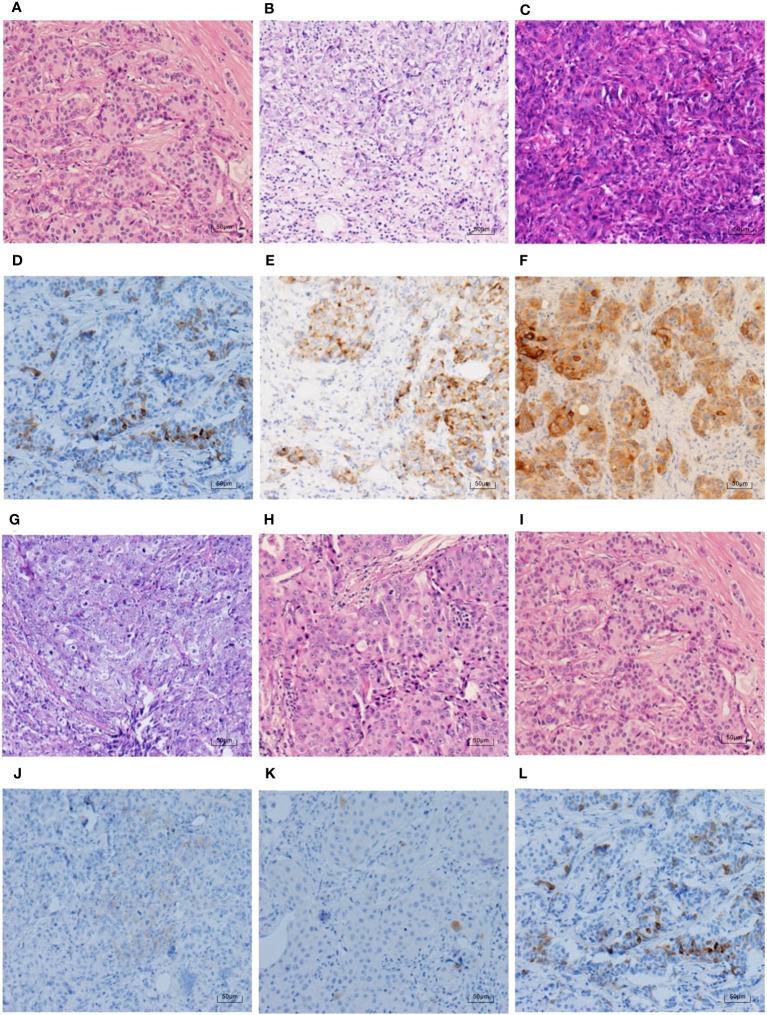
Syn IHC staining and corresponding HE staining in TNBC (100×). **(A–C, G–I)** show the HE staining in the same case as **(D–F, J–L)**, respectively. **(H)** shows the NST with apocrine differentiation, and the rest are IBC-NST. The percentages of Syn positive tumour cells in **(D–F)** are ranked from the lowest to the highest, which are 5%, 25%, and 95%. **(J–L)** rank the intensity of Syn staining from weak to strong, in order of weak positive, medium positive, and strong positive.

**Figure 3:**
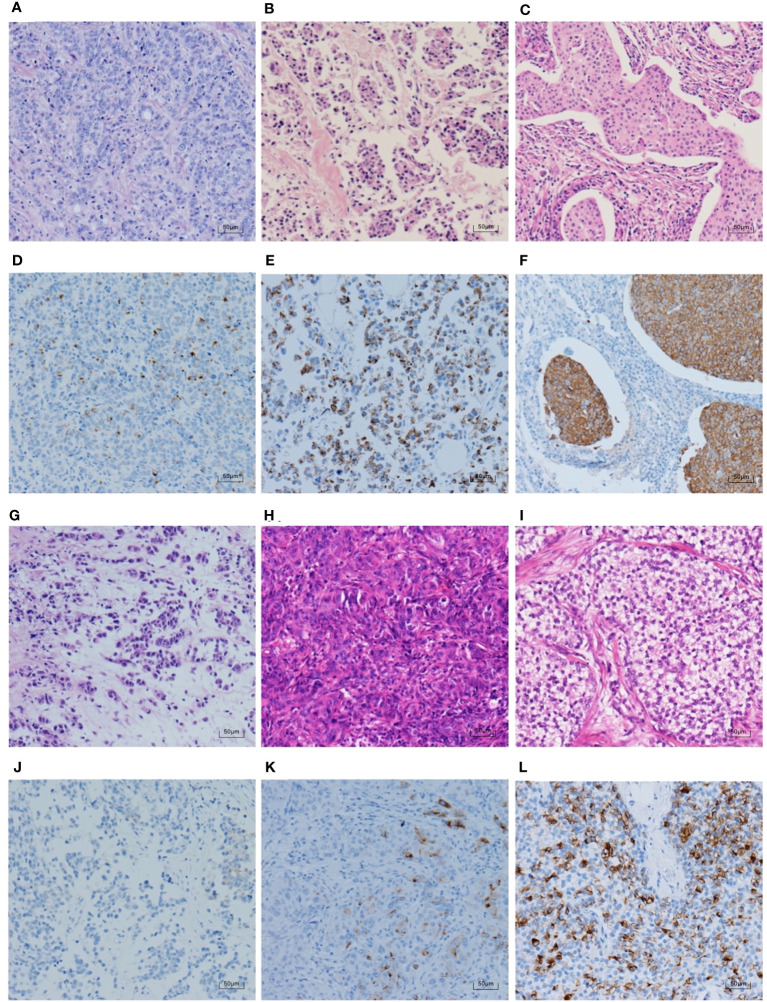
CgA IHC staining and corresponding HE staining in TNBC (100×). **(A–C, G–I)** show the HE staining in the same case as **(D–F, J–L)**, respectively. **(B)** shows invasive micropapillary carcinoma, and the rest are IBC-NST. The percentages of CgA positive tumour cells in **(D–F)** are ranked from the lowest to the highest, which are 5%, 60%, and 95%. **(J–L)** rank the intensity of CgA staining from weak to strong, in order of weak positive, medium positive, and strong positive.

**Figure 4:**
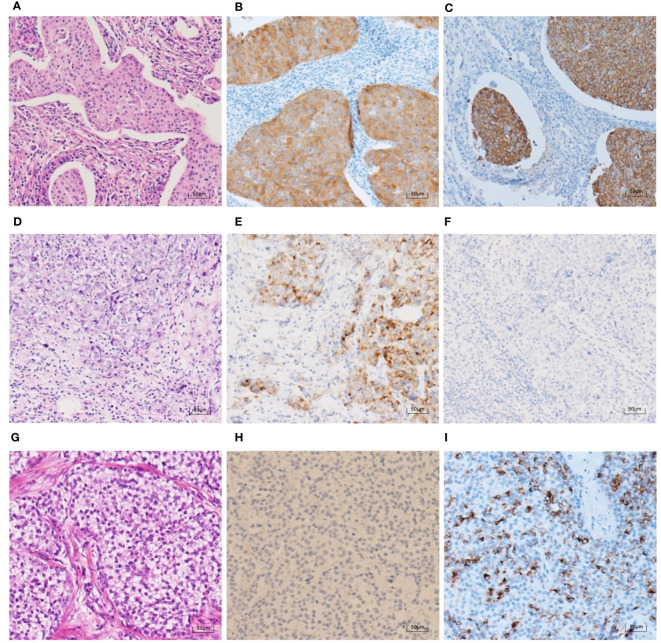
HE staining and NE marker IHC staining in 3 TNBC cases (100×). **(A–C)** An IBC-NST case with both positive Syn expression (70% medium-strong positive) and positive CgA expression (90% strong positive). **(D–F)** An IBC-NST case with positive Syn expression (25% medium-strong positive) and negative CgA. **(G–I)** An IBC-NST case with negative Syn and positive CgA expression (60% strong positive).

To further clarify the pathological features of the TNBC cases with positive expression of NE markers, we reviewed the pathological sections of the 30 cases according to the newly published WHO Classification in 2019. The histological types are shown in **Table 6**. Most of the cases (23 cases, 76.7%) were IBC-NST. Other histological types included six cases of NST with apocrine differentiation and one case of invasive micropapillary carcinoma. Syn and CgA IHC staining images and the corresponding hematoxylin & eosin (HE) staining images are shown in **Figures 2**–**4**. Microscopically, the tumor cells were clear and had different degrees of atypia. Pathological mitoses were observed. Most cells were arranged in nests or solid sheets, with some having papillary structures. Syn and CgA IHC images showed that the cytoplasm of some tumor cells was colored to varying degrees, showing scattered or large patchy light brown to dark brown staining.

**Table 6:** Histological types of the 30 TNBC cases with positive expression of NE markers.

Histological type	N (%)
IBC-NST	23 (76.7)
NST with apocrine differentiation	6 (20.0)
Invasive micropapillary carcinoma	1 (3.3)

### 3.5 ATRX, MGMT, SSTR2, and PD-L1 expression in NE marker-positive TNBC cases

To further explore the prognosis and potential treatment options of NE marker-positive TNBC, we conducted IHC staining of ATRX, MGMT, SSTR2, and PD-L1 in the 30 TNBC samples with positive NE marker expression. The results are shown in **Tables 7****–****9**.

**Table 7:** IHC staining results of ATRX and MGMT in the NE marker-positive TNBC cases.

		N (%)	
		ATRX	MGMT
Intensity of staining	Weak positive	6 (20.0)	10 (33.3)
	Medium positive	16 (53.3)	9 (30.0)
	Strong positive	4 (13.3)	3 (10.0)
Percentage of positive tumour cells	1%-20%	4 (13.3)	13 (43.3)
	21%-40%	6 (20.0)	4 (13.3)
	41%-60%	2 (6.7)	1 (3.3)
	61%-80%	8 (26.7)	3 (10.0)
	81%-100%	6 (20.0)	1 (3.3)
	Negative	4 (13.3)	8 (26.7)

**Table 8:** IHC staining results of SSTR2 in the NE marker-positive TNBC cases.

IHC results of SSTR2	N (%)
0	16 (53.3)
1+	5 (16.7)
2+	5 (16.7)
3+	4 (13.3)

**Table 9:** IHC staining results of PD-L1 (22C3) in the NE marker-positive TNBC cases.

IHC results of PD-L1 (22C3)		N (%)
TPS%	0	20 (66.7)
	1-3	7 (23.3)
	30	2 (6.7)
	40	1 (3.3)
CPS	0	12 (40.0)
	1-5	13 (43.3)
	6-10	1 (3.3)
	20	1 (3.3)
	31-40	3 (10.0)

Among the 30 NE marker-positive TNBC cases, four cases (13.3%) and eight cases (26.7%) were negative for ATRX and MGMT IHC staining, respectively. More than half of the cases (53.3%) were medium-positive and eight cases (26.7%) were 61%–80% positive for ATRX IHC staining. Ten cases (33.3%) were weak-positive and 13 cases (43.3%) were 1%–20% positive for MGMT IHC staining.

**Figure 5:**
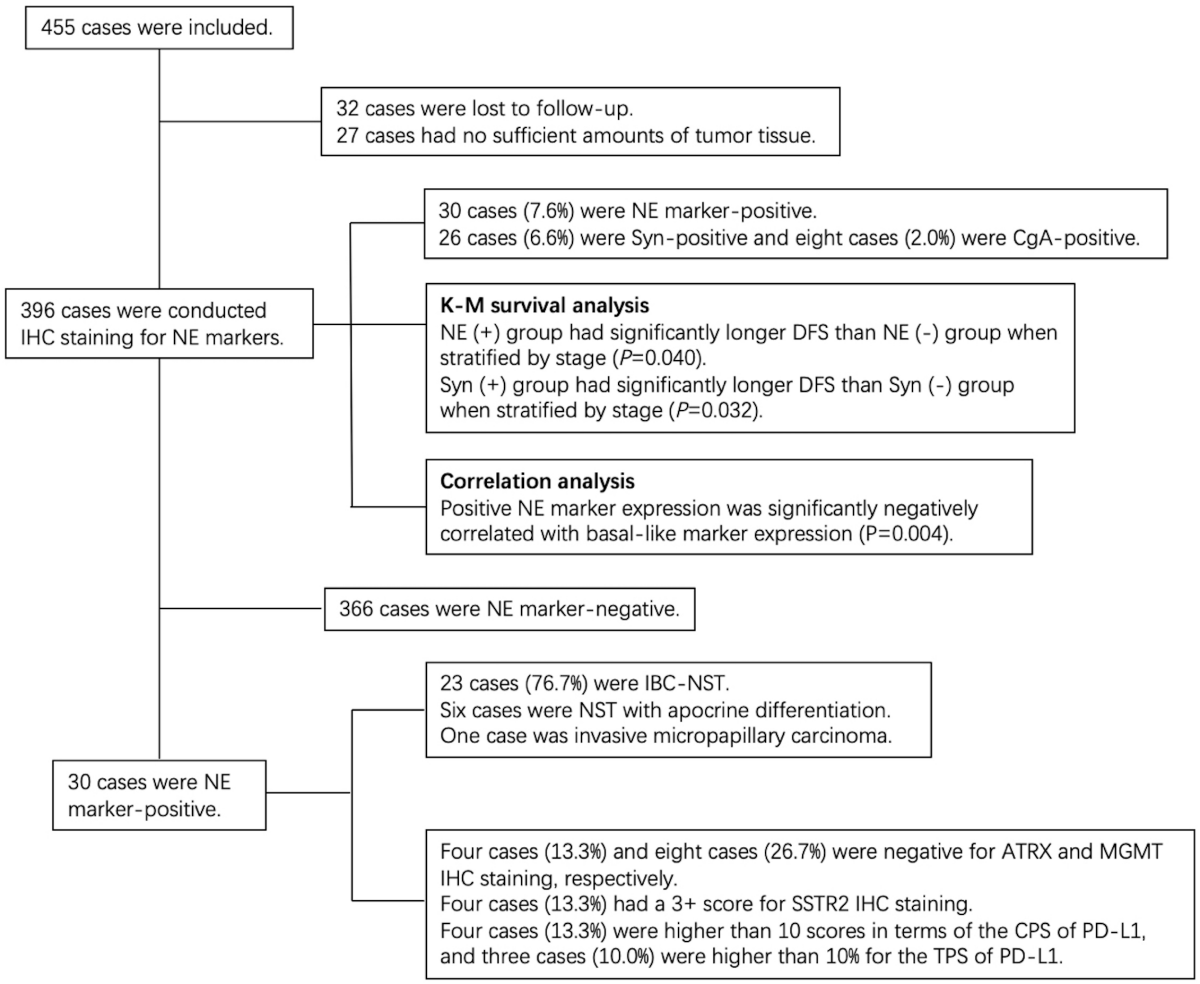
The summary of the results in this study.

Furthermore, four of the 30 NE marker-positive TNBC cases (13.3%) had a 3+ score for SSTR2 IHC staining. In addition, more than half of the cases (53.3%) had a 0 score (negative SSTR2 IHC staining). Four cases (13.3%) among the 30 TNBCs were higher than 10 scores in terms of the CPS of PD-L1, and three cases (10.0%) were higher than 10% for the TPS of PD-L1. The summary of the results of our study is presented in **Figure 5**.

## 4 Discussion

Because of the highly malignant nature, poor prognosis, and high heterogeneity associated with TNBC, researchers have been committed to identifying prognostic biomarkers and therapeutic targets specific to this disease. A large retrospective cohort study that collected data from nearly 160,000 breast cancer patients from the National Cancer Institute in 2017 showed that TNBC patients had a worse prognosis than non-TNBC patients of the same stage, adjusted for age, race, tumor stage, surgery, and radiation. These findings confirmed the importance of introducing biomarkers to the traditional TNM staging to help predict breast cancer patient prognosis, as well as the necessity of further exploring TNBC-related issues (32).

To our knowledge, our study is the first and the largest retrospective cohort study to explore the prognostic significance of NE markers in TNBC. We aimed to provide new biomarkers that can help predict TNBC patient survival and assist clinicians with treatment decisions.

Among the 396 TNBC patients included in this study, positive NE marker expression was observed in 30 cases (7.6%). 20 cases had <10% of tumor cells positive for NE markers. Eight cases had 10%–89% of tumor cells positive for NE markers. Two cases had ≥90% of tumor cells positive for NE markers. The WHO Classification of Tumours of the Breast in 2019 suggested that cancers with more than 90% NEN pattern should be classified as neuroendocrine tumor or NEC, while cancers with less than 10% should be classified as invasive NST or other types. Cancers with 10% to 90% NEN pattern should be classified as mixed invasive (NST or other special type) and NEC, and the percentage of NE component should be reported (8). According to the WHO Classification in 2019, two of these 30 cases were classified as NENs. Seven cases were classified as mixed invasive NST and NEC and one case were classified as mixed invasive micropapillary carcinoma and NEC. According to the 2012 WHO Classification of Tumours of the Breast, NED can be detected in approximately 30% of IBC-NST cases and in some special types (especially mucinous carcinoma) by IHC staining (6). However, because of different definitions, the incidence of NENs in all types of breast cancer has been previously reported to be as low as < 0.1% to as high as nearly 20% (9, 11, 13, 15, 16). For the TNBC subtype in our cohort, the percentage of cases with positive NE marker expression (7.6%) was lower than the incidence of NED in all breast cancers (30%) indicated by the 2012 WHO Classification of Tumours. Additionally, the incidence of NENs was very low according to the 2019 WHO Classification of Tumours definition. Only two cases had more than 90% of NE marker-positive tumor cells among the 396 TNBC patients. These results are also consistent with the previous findings that NED is significantly correlated with ER and PR expression (13, 33).

According to the K-M survival analysis for the TNBC patients stratified by stage in our study, we found that those with positive NE marker expression had higher DFS rates than those without NE marker expression in the same stage. To our knowledge, no previous cohort study has reported on the prognostic effect of NE markers in TNBC patients. In general, sufficient studies have confirmed that NED in breast cancer implies a poor prognosis (4, 9–11). Actually, our result does not conflict with the conclusions of previous studies. The objects of previous studies were breast cancer patients, which were dominated by hormone-positive cases, and the numbers of TNBC cases were small. While our study focused on TNBC, a breast cancer subtype with a worse prognosis relative to others. Therefore, we speculated that the influence of NE markers on prognosis of breast cancer patients might be related to their hormonal status.

In addition, our study showed that positive Syn expression has a better prognostic significance than CgA. Previous work has concluded that NED can be confirmed using IHC stains for several markers, including Syn, and CgA. Each marker has different degrees of specificity and sensitivity: Syn is highly sensitive, but not entirely specific. CgA is highly specific, but less sensitive than Syn (34). In our study, 6.1% and 1.9% of TNBC cases showed positive IHC staining for Syn and CgA, respectively. Positive Syn expression significantly prolongated the DFS in TNBC patients compared with Syn-negative TNBC patients at the same stage, but no significant difference in DFS was found for CgA expression. Our study concludes that Syn positivity in TNBC was higher than that of CgA, and the prognostic significance of Syn was greater than that of CgA for TNBC patients. The results of our study may provide guidance for clinical identification of TNBC patients with better prognoses.

Furthermore, we observed a significant negative correlation between NE marker expression and basal-like marker expression in our cases. Previous literature suggests that positive expression of basal-like markers can be used as adverse prognostic factors for TNBC (35–37). These conclusions are consistent with our results of positive NE marker expression being associated with a better prognosis in TNBC, as well as the observed negative correlation between NE marker and basal-like marker positivity.

After further exploring the 30 TNBC cases with positive NE marker expression, we found that 13.3% of these cases were negative for ATRX expression, indicating poor prognosis (23, 24). 26.7% of these cases were negative for MGMT expression, which may response to temozolomide (25–27). Additionally, 13.3% of these cases had a 3+ score for SSTR2 IHC staining, suggesting poor prognosis but also the potential to be treated with somatostatin analogues (28). The TPS of PD-L1 was higher than 10% in 10.0% of these 30 cases, meaning that a small percentage of the NE marker-positive TNBC patients may benefit from immunotherapy (29, 30). However, because of the low levels of NE marker expression in TNBC and the small number of cases included, further investigation is warranted regarding the positive rate of IHC staining for these markers and their prognostic and therapeutic implications. A meta-analysis involving 49,425 cancer patients suggested that immune checkpoint inhibitors (ICIs) may increase the possibility of complete remissions compared to control treatments (38). Nevertheless, only a subset of breast cancer patients benefit from ICIs, necessitating exploration into reliable predictors of response. A review discussing potential predictors of response to ICIs in TNBC indicated that PD-L1 could serve as an important marker for selecting TNBC patients who would derive greater clinical benefits from immunotherapy. However, relying solely on PD-L1 assessment might exclude certain TNBC patients who could potentially respond to immunotherapy. More efforts should be devoted to evaluating novel biomarkers for predicting response to ICIs in TNBC patients, such as PD-L1 expression, tumor mutational burden, microsatellite instability status, etc. (39) Recent clinical trials have demonstrated the benefits of various immune-based combinations for metastatic TNBC patients; thus chemoimmunotherapy has been approved as a new first-line treatment option for metastatic TNBC patients with elevated CPS or PD-L1 overexpression. Several clinical studies potentially modifying the landscape of first-line treatment options for TNBC are still ongoing (40).

This study has some limitations. Differences in the time from disease onset to when surgical treatment was administered, the surgical methods received, and the adjuvant therapies became confounding factors that affected our prognostic evaluation. We reduced the effect of the different treatment regimens on prognosis by excluding patients who received neoadjuvant therapy.

In conclusion, expression of NE markers, especially Syn, in TNBC indicated a better prognosis compared with cases negative for NE marker expression at the same stage. There was a significant negative correlation between NE marker expression and basal-like marker expression in TNBC. The use of NE markers as biomarkers for predicting TNBC patient prognosis requires further verification. Additionally, we found that a small number of TNBC cases with positive NE marker expression also showed positive IHC staining for PD-L1 and SSTR2, suggesting that these patients may potentially benefit from immunotherapy or somatostatin analogue treatment.

## Data availability statement

The raw data supporting the conclusions of this article will be made available by the authors, without undue reservation.

## Ethics statement

The studies involving humans were approved by the Ethics Committee of Peking Union Medical College Hospital. The studies were conducted in accordance with the local legislation and institutional requirements. The human samples used in this study were acquired from primarily isolated as part of your previous study for which ethical approval was obtained. Written informed consent for participation was not required from the participants or the participants’ legal guardians/next of kin in accordance with the national legislation and institutional requirements.

## Author contributions

CX, ZL, and XR contributed to the conception and design of the study. SS, JP, LC, and JY acquired and organized the data for the work. CX performed the statistical analysis and wrote the first draft of the manuscript. XR wrote sections of the manuscript. All authors contributed to the article and approved the submitted version.
